# P53 Expression in benign Breast Disease Development: A Systematic Review

**DOI:** 10.31557/APJCP.2020.21.9.2485

**Published:** 2020-09

**Authors:** Rafaela Soares Senra da Costa, Ilce Ferreira da Silva

**Affiliations:** *National School of Public Health Sergio Arouca, Oswaldo Cruz Foundation, Rio de Janeiro, Brazil. *

**Keywords:** Breast diseases, genetic polymorphism, epidemiology, public health

## Abstract

**Background:**

Benign breast disease (BBD) is one of main breast cancer risk factors. Dysfunctions on p53 protein, which has a genome protective role, have been related to breast cancer developments. However, its role on BBD development is still unclear.

**Methods:**

A systematic review of literature was proceeded according to PRISMA-P guidelines. PubMed, BVS, MEDLINE and Scholar Google were used as databases, complemented by a manual search in articles references. Articles searches were conducted from May to July 2019 and publications in English, Spanish and Portuguese were selected. P53 expression was set as outcome among women with BBD and were included only articles with good quality according STROBE tools. Data concerning p53 expression frequencies were independently extracted by two review authors, and eligible articles were synthesized.

**Results:**

From 12 studies selected for this review, the majority analyzed p53 expression in non-proliferative lesions and general p53 expressions ranged from 0 to 100%. P53 expression was more frequently observed in cases series studies (91.7%) and in studies conducted in Occidental Europe (41.7%). P53 expression was more frequent among tissues with fibrocystic disease (22.5%) and fibroadenoma (22.5%).

**Conclusion:**

When compared with all breast tissues types, benign breast disease corresponds to 34.39% of p53 expression. Second outcomes were not evaluated because the heterogeneity observed in selected studies. In addition, more studies considering ethnicity and benign breast disease classification should also be considered for further analysis.

## Introduction

Benign breast disease (BBD) are highly frequent in female population, corresponding to 80% of all breast lesions (Ajao, 1979; Guray and Sahin, 2006). BBD encompass a large and heterogeneous group of lesions detectable through microscopic findings and imaging tests (Page and Dupont, 1993). This type of lesion is more incident in the second decade of life, stagnating in the fourth decade (Guray and Sahin, 2006). BBD symptomatology include breast pain, presence of nodules and papillary discharge (Santen, 2000). Estimating BBD frequencies according to histological group is considered difficult, since some women with BBD not necessarily need treatment or specialized medical attention for diagnosis (Goehring and Morabia, 1997); and also because most women have multiple types of lesions of different histological groups (Santen, 2000). 

BBD are divided according to their risk for breast cancer as non-proliferative, proliferative without atypias and atypical hyperplasias (College of American Pathologists, 1986). Non-proliferative lesions include cysts, papillary apocrine changes, epithelial calcifications, hyperplasias (between two and four layers of epithelial cells within ducts), and fibroadenomas. Breast cancer risks for such lesions range from 0.80 (95%CI 0.58-1.11) to 1.60 (95%CI 0.99-2.58) (Dyrstad et al., 2015). Proliferative lesions without atypia included moderate or florid hyperplasia, intraductal papilloma, and sclerosing adenosis; with estimated breast cancer risks varying from 1.20 (95%CI 0.47-3.04) to 7.26 (95%CI 2.17-24.27); (Dyrstad et al., 2015). Atypical hyperplasias constitute a major breast cancer risk group (OR:2.10;95%CI 1.12-3.95 to OR:25.20;95%CI 3.68-172.67), comprising proliferative lesions that have some characteristics of carcinoma in situ, as well as atypical ductal hyperplasia and atypical lobular hyperplasias (Page et al., 1978, 1985; Dupont and Page, 1985). 

P53 protein is encoded by TP53 gene, exerting a genome protective function by regulating cell cycle in G1/S at checkpoint for DNA repair (Levine, 1997), or through apoptosis induction in genetically damaged cells (Agarwal et al., 1998). Mutations and genetic polymorphisms can alter p53 protein, leading to imbalances in main gene functions (Pharoah et al., 1999). Somatic mutations or sub-regulation of p53 protein may contribute to breast cancer development in addition to being associated with a more severe prognosis (Oliveira et al., 2005). These mutations alter p53 protein standard structure, which triggers the inhibition of DNA repair mechanisms inducing to apoptosis (Bourdon et al., 2003; Bourdon, 2007) . 

Literature supports that cell accumulation of p53 inactive form exponentially increases expression of mutant p53 proteins, with a longer half-life. Consequently, accumulation of p53 in tumor tissues is directly related to presence of p53 mutations (Dowell et al., 1994; Casey et al., 1996). When it occurs, cells with mutated p53 protein have major probability to accumulate mutations and additional chromosomal rearranges. This condition may assist in proliferation of mutated DNA cells, modulating the BBD evolving process into breast cancer lesion (Júnior et al., 2002; Bourdon et al., 2003; Oliveira et al., 2005; Bourdon, 2007). Given the importance of functionality of p53 protein in cell growth process and, consequently, BBD development, the present systematic review study aims to summarize studies that evaluated the pattern of p53 protein expression among women with BBD. 

## Material and Methods

This systematic review of literature was registered in International Prospective Register of Systematic Reviews (PROSPERO number CRD42017073720). Preferred Reporting Items for Systematic reviews and Meta-analyses for Protocols (PRISMA-P Group, 2015) statement studies guided studies selection. Eligibility criteria definitions included Participants, Interventions, Comparators, Outcomes, Timing and Study designs (PICOTS). Women over 17 years old diagnosed with benign breast diseases (BBD) were considered as population. Interventions were considered as any type of breast biopsy, resulting in BBD confirmation by histopathology report. Outcome was defined as p53 expression in lesion tissue of women diagnosed with BBD, while p53 protein expression in women with normal breast tissue was considered as reference. Breast cancer cases were also considered in cohort and case controls studies, when evaluated p53 expression among women diagnosed only with benign breast disease tissue. There was no restriction based on study design, neither on the length of patient follow-up time for longitudinal studies. Only observational studies were selected for the present review.

**Figure 1:**
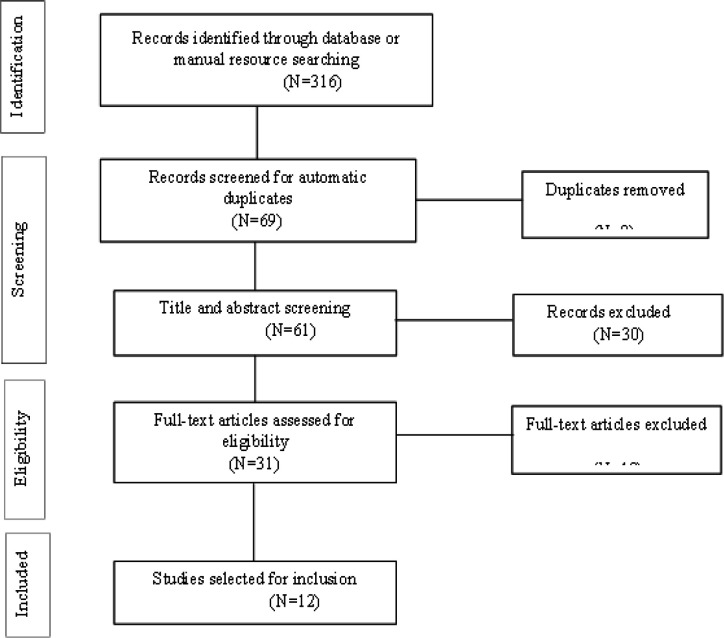
Flow Diagram of Articles Inclusion According to PRISMA (2018)

Systematic search strategy was used including “benign breast disease” AND (“TP53” OR “p53”) terms and their variations in PubMed, Biblioteca Virtual em Saúde (BVS), Medline and Scholar Google databases. Besides, manual searches were implemented to increase available literature and ensure that all eligible studies were selected. Articles reported in English, Spanish, and Portuguese languages were included. Exclusion criteria were applied when p53 expression in presence of TP53 mutations or somatic mutations were evaluated among women diagnosed with present or past breast cancer history. Selected articles were managed in Zotero Standalone software (version 4.0.29.17) to import references and abstracts from electronic databases. Zotero software was used to remove duplicates and facilitate the screening process. Also, all titles and abstracts were assessed against inclusion criteria and reasons for articles inclusion or exclusion are documented and present in a flow diagram of articles inclusion according to PRISMA (Figure 1). 

Data was extracted by two trained researchers(authors), who had independently carried out records selection, discrepancies about data extraction were resolved by discussion and consensus. For each article selected for this review, data extraction included authors, publication year, population country, study design, quantitative population, age of population (median or age range), p53 analysis type and p53 expression percentage. Primary outcome was defined as the frequency of p53 expression among women diagnosed with any type of BBD, and among controls. P53 expression, according to germinal and somatic mutations, were considered as secondary outcomes. Type of p53 expression analysis, menopausal status (if available), and ethnicity were considered confounding variables. Bias risks for each study were analyzed using the Cochrane Collaboration tool for assessing the risk of bias (Higgins et al., 2011). Low-quality studies, according Strengthening the Reporting of Observational studies in Epidemiology (STROBE) instruments, were discarded. Data analysis was performed through the findings’ frequencies describing p53 expression according to BBD, in the included studies. So, p53 expression frequencies were expressed in two forms. Firstly, in comparison only among benign breast disease types showed in selected studies. Thereafter, p53 expression percentage observed in selected studies was demonstrated among tissues diagnosed as normal, benign breast disease, borderline and breast cancer.

**Figure 2:**
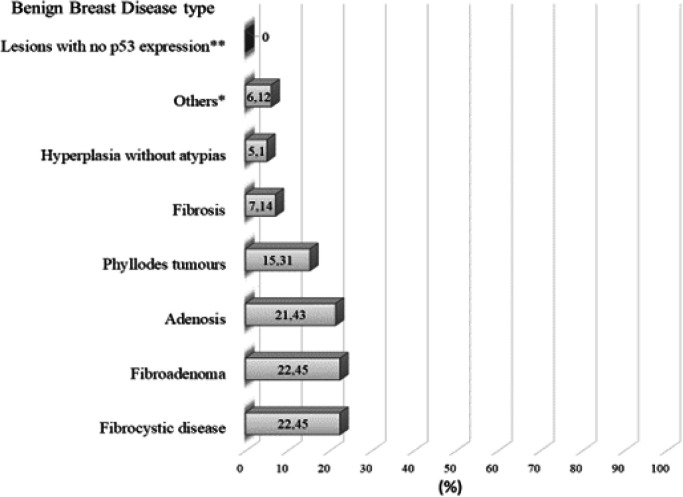
P53 Expression among benign Breast Disease Types Observed in Included Studies. **, Lactating adenoma, duct ectasia, mastitis, radial scar, ductal hyperplasia with atypias and lobular hyperplasia with atypias; *, Lesions with one case with p53 expression: inflammation, intraductal papilloma, adenoma, sclerosing adenosis, and complex fibroadenoma

**Figure 3:**
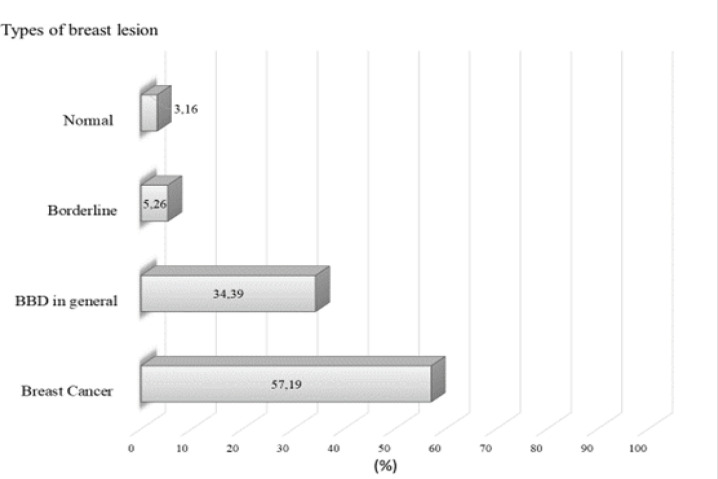
P53 Expression According to Any Tissue Type Observed among Breast Biopsies of Included Studies

**Table 1:** Included Studies Summarize

Author, year	Local	Study design	Population	Age (years)	p53 expression (%)
Younes et al, 1995	USA	Cohort	Follow up: 60 months 23 breast cancer 248 benign breast disease: 01 lactating adenoma 30 fibrosis 09 inflammation 04 intraductal papilloma 05 duct ectasia 105 fibrocystic disease 71 fibroadenoma 23 normal tissue	−	Breast cancer= 30.0% Benign breast disease=16.0% Lactating adenoma: 0% Fibrosis: 23.0% Inflammation: 11.0% Intraductal papilloma: 0% Duct ectasia: 0% Fibrocystic disease: 8.0% Fibroadenoma: 30% Normal tissue=9.0%
Sirotkovic-Skerlev et al, 2005	Croatia	Case series	85 breast cancer 51 benign breast disease	−	Breast cancer = 34.0% Benign breast disease=19.6%
Eriksson et al, 1994	Germany	Case series	340 breast cancer 109 in situ 89 benign breast disease: 09 fibroadenoma 04 intraductal papilloma 02 adenoma of nipple 02 tubular adenoma 07 fibrocystic disease 04 sclerosing adenosis 03 mastitis 04 radial scar 54 intraductal hyperplasia whitout atypia 21 intraductal hyperplasia with atypia 03 lobular hyperplasia with atypia 05 normal tissues	−	Breast cancer= 22.9% In situ=11.9% Benign breast disease= 0% Normal tissue= 0%
Ioakim-Liossi et al, 1998	Greece	Case series	60 breast cancer 20 benign breast disease: 15 fibroadenoma 05 fibrocystic change	−	Breast cancer= 45.0% Benign breast disease= 0%
Feankis et al, 1999	UK	Case series	57 phyllodes tumours: 13 malignant 17 borderline 27 benign	58 to 76	Malignant=39.0% Borderline=18.0% Benign=0%
Kandel et al, 2000	Canada	Case series	42 benign breast disease: 22 fibrocystic change 08 adenosis 02 fibroadenomas 08 hyperplasia whitout atypias 02 normal tissues	−	Fibrocystic change=22.7% Adenosis=50.0% Fibroadenomas= 0% Hyperplasia whithout atypias=25.0% Normal tissue=0%
Niezabitowski et al, 2001	Poland	Case series	117 phyllodes tumor 42 malignant 23 borderline 52 benign	−	Malignant: 59.5% Borderline: 52.2% Benign: 28.8%
Ryška et al, 2001	USA	Case series	06 benign breast disease with multinucleated stromal giant cells: 01 adenomyoephitelioma 01 adenosis 01 intraductal papilloma 01 complex fibroadenoma 01 adenosis with focal atypic ductal hyperplasia 01 fibroadenoma with tubular pattern	37 to 70	Benign breast disease=83.3% Adenomyoephitelioma, adenosis, intraductal papilloma, complex fibroadenomas, adenosis with focal atypic ductal hyperplasia= 0% Fibroadenoma with tubular pattern = 100%
Selim et al, 2002	UK	Case series	82 benign breast disease: 18 apocrine adenosis 64 apocrine metaplasia	−	Apocrine adenosis=27.8% Apocrine metaplasia=1.6%
Herbert et al, 2002	Israel	Case series	24 hamartomas	30 to 78	0%
Angele et al, 2004	UK and France	Case series	18 breast cancer 15 benign breast disease: 08 sclerosing adenosis 07 breast papillomas 08 normal breast tissues	−	Breast cancer=44.4% Benign breast disease=0% Normal tissues=0%
Khalifeh et al, 2008	USA	Case series	10 microglandular adenosis	30 to 70	100%

## Results

Initial search strategy in databases and manual resource searching identified 316 records. At screening duplicates were removed while 61 records were screened by title and abstract. Afterwards, 31 full-text records were assessed for eligibility, being 19 of them excluded in this phase. A total of 12 studies were selected for this review (Figure 1).

All 12 included studies were summarized in Table 1. Most studies were proceeded in Occidental Europe (41.7%), followed by North America (33.3%), Oriental Europe (16.7%) and Asia (8.3%). Case series was the most frequent study design (91.7%), followed by 1 cohort study. Among female breast tissues present on selected studies, were observed normal tissues, benign breast disease (BBD) tissues, carcinoma in situ tissues and breast cancer tissues. Participants age ranged from 30 to 78 years. All selected studies evaluated p53 expression in non-proliferative lesions, which included lesions such as lactating adenoma, duct ectasia, hyperplasia without atypias, apocrine metaplasia, inflammation, fibrocystic disease, fibrosis, fibroadenoma, adenosis, apocrine adenosis and microglandular adenosis. On the other hand, p53 expression was also observed in proliferative without atypias BBD group, which included papillomas, phyllodes tumours and sclerosing adenosis. Few studies included atypic hyperplasias group, which included isolated intraductal hyperplasia with atypia, adenosis with focal atypic ductal hyperplasia and lobular hyperplasia. 

Paraffin-embedded biopsy was the matrix of p53 expression analysis in all included studies. Monoclonal antibodies’ immunohistochemistry technique was employed in all studies selected to determinate p53 expression. Additionally, two studies analyzed amino acid changes in TP53 gene. Kandel et al., (2000), analyzed amino acid changes in exons 4 to 10 by polymerase chain reaction-single-strand conformation polymorphism (PCR-SSCP) genotyping technique. Kandel et al., (2000) in a case series included 42 breast samples composed by 2 normal tissues, 22 fibrocystic change tissues, 8 adenosis tissues, 2 fibroadenomas tissues, and 8 hyperplasias without atypias tissues. Authors observed different p53 expressions according to mutation presence. When considered only p53 expression, was observed no expression among normal and fibroadenomas tissues (0%), followed by fibrocystic change (22.7%), hyperplasia without atypia tissues (25.0%) and adenosis tissues (50.0%); as described in Table 1. 

PCR-SSCP considered mutations with aminoacid changes in exon 4 (codons72,76 and 110), exon 5 (codons133,135,175 and 178), exon 7 (codons 227,244 and 245) and exon 9 (only codon 325); sequence changes without amino acid changes as silent changes in exon 4 (codons 74 and 111), exon 6 (only codon 217), exon 7 (codons 239 and 226); and intronic changes in intron 6 (nr2 13466), intron 7 (nr 14114) and intron 9 (nr 14766). When associated p53 expression with presence of TP53 mutation, Kandel et al., (2000) observed different p53 expression than described before. Lowest p53 expression were observed among adenosis tissues (12.5%), followed by hyperplasia without atypias tissues (37.5%), fibrocystic change tissues (40.9%), fibroadenomas tissues (50.0%) and normal tissues (100%).

Figure 2 shows p53 mean expression among benign breast disease observed in selected studies. Among selected studies, p53 expression was not observed in lactating adenomas, duct ectasia, mastitis, radial scar, ductal hyperplasia with atypias and lobular hyperplasia with atypias. P53 expression was around 1.02% among inflammation, intraductal papilloma, adenoma, sclerosing adenosis and complex fibroadenoma. Fibrocystic disease and simple fibroadenoma showed the highest p53 expression frequency (around 22.4%). 

P53 expression according to tissue type is showed in Figure 3. Normal tissues presented the lowest p53 expression (3.16%) among all selected studies in this review. Borderline lesions were considered only for phyllodes tumours considered as borderline by authors, and showed 5.26% of p53 expression. Considering p53 expression among BBD in general, a frequency of 34.39% was observed. Among all types of breast lesion included in the studies, breast cancer showed the major p53 expression (57.19%). 

## Discussion

P53 protein exerts a genome protective function, acting on G1/S cell cycle regulation at DNA repair checkpoint (Levine, 1997), or by inducing apoptosis in genetically damaged cells (Agarwal et al., 1998). In presence of lesions, it is a function of p53, through a cascade of reactions, to prevent this cell from mitosis and complete cell division. It can occur as a consequence the correction of mutation through activation of repair protein or induction to apoptosis (Rivoire et al., 2001).

Cells with a p53 mutated, does not occur cell cycle stop, which makes them unstable and with a tendency to accumulate additional mutations and chromosomal rearrangements, probably leading to mutated cells proliferation, with subsequent formation of neoplasms and, in breast cancer cases, more severe prognosis (Bourdon et al., 2003; Oliveira et al., 2005; Bourdon, 2007). It is also known that p53 inactive form accumulation within cells exponentially increases mutant p53 expression as a consequence of increased DNA damage. Thus, p53 accumulation in tumor tissues is directly related to presence of mutation in p53 protein (Dowell et al., 1994; Casey et al., 1996).

To the best of our knowledge, this is the first systematic review to investigate p53 expression pattern among women diagnosed with benign breast disease. P53 expression showed a great range among benign breast disease. Evidences observed in these selected studies, showed that the p53 analyses among women with BBD are still scarce. For example, atypic hyperplasias group was poorly represented in selected studies, regardless their high-risk for breast cancer development (Eriksson et al., 1994; Millikan et al., 1995; Wells et al., 1995; Younes et al., 1995; Ioakim-Liossi et al., 1998; Feakins et al., 1999; Kandel et al., 2000; Niezabitowski et al., 2001; Ryška et al., 2001; Herbert et al., 2002; Selim et al., 2002; Angèle et al., 2004; Sirotkovic-Skerlev et al., 2005; Khalifeh et al., 2008; Shin et al., 2009).

A limitation observed was that not all studies described any benign breast disease type or group lesion (Eriksson et al., 1994; Millikan et al., 1995; Ioakim-Liossi et al., 1998; Ryška et al., 2001; Herbert et al., 2002; Angèle et al., 2004; Sirotkovic-Skerlev et al., 2005; Khalifeh et al., 2008; Shin et al., 2009), what implicates in difficulties in summarizing and comparing the results. On the other hand, 2 studies (16.7%) observed p53 expression only among women with phyllodes tumors (Feakins et al., 1999; Niezabitowski et al., 2001), showing great differences between p53 expression in totally benign, borderline and malignant lesions. 

Such distinctions may be a reflection of biopsy techniques and different forms of BBD classification according to pathologist’s perspective, which can lead to differences in the findings (O’Flynn et al., 2010; Simpson et al., 2010). These p53 expression ranges can be observed in differential diagnosis performed on lesions classified as atypical hyperplasias and types of carcinomas in situ. However, BBD classification may differ according criteria used by pathologists to classify them, depending on lesion size, breast cancer risk, lesion histopathological behavior and nomenclatures used (Dupont and Page, 1985; Fitzgibbons et al., 1998).

Added to all BBD classification that could implicate in results observed in this review, most lesions with p53 expression analysed were classical classified as non-proliferative according to Dupont and Page (1985). Also, there was not a great studies quantity realized in different geographical regions, which could imply comparative analysis of p53 expression according to skin color. In other words, number of selected studies implied in an impossibility of another outcomes comparative analysis in this systematic review. 

The present systematic review was not expanded into a meta-analysis because data was not comparable (homogeneous) across the included studies. According to Kelley and Chung (2017) inappropriate pooling of heterogeneous studies could result in misleading statistical results, what decreases meta-analysis quality. So only homogeneous data should be pooled and statistical analysis of pooled data would be difficult for reason cited in this systematic review, as differences among study designs, and differences on benign breast disease classification (Cheung et al., 2012; Cheung and Vijayakumar, 2016; Kelley and Chung, 2017). Despite that, the present systematic review could be the first step to shed light on BBD biological mechanism, as well as to highlight the need of more studies about the association between p53 expression and BBD, with analytical designs, greater sample size, and more homogeneous grouping of BBD.

 This systematic review selected 12 studies who observed p53 expression among women diagnosed with benign breast disease. Most of selected studies analyzed p53 expression among breast tissues with non-proliferative lesions. When compared with all breast tissues types, benign breast disease corresponds to 34.39% of p53 expression. BBD with major breast cancer risk was poorly represented among selected studies. Second outcomes were not evaluated because the heterogeneity observed in selected studies. Given the importance of p53 protein in the breast cancer development, studies related to p53 expression among benign breast disease are very important; especially in hyperplasic lesions that show major risk for breast cancer progression. In addition, more studies considering ethnicity and benign breast disease classification should also be considered for further analysis. 
